# Cephalexin Model-Informed Dosing Recommendations for Severe Infections: A Pooled Pharmacokinetic Analysis From Infancy to Adulthood

**DOI:** 10.1093/ofid/ofag611

**Published:** 2026-09-26

**Authors:** Andrew S Haynes, Daniel Gonzalez, Kevin Messacar, Julie Autmizguine, Amanda Gwee, Richard Everts, Peter Anderson

**Affiliations:** Department of Pediatrics, Section of Pediatric Infectious Diseases, Children's Hospital Colorado, Aurora, Colorado, USA; School of Medicine, University of Colorado Anschutz Medical Campus, Aurora, Colorado, USA; Division of Clinical Pharmacology, Department of Medicine, Duke University School of Medicine, Durham, North Carolina, USA; Duke Clinical Research Institute, Durham, North Carolina, USA; Department of Pediatrics, Section of Pediatric Infectious Diseases, Children's Hospital Colorado, Aurora, Colorado, USA; School of Medicine, University of Colorado Anschutz Medical Campus, Aurora, Colorado, USA; Department of Pediatrics, Section of Pediatric Infectious Diseases, Sainte-Justine University Hospital, Montreal, Canada; Azrieli Sainte-Justine Research Center, Montréal, Canada; Department of Pharmacology and Physiology, Université de Montréal, Montréal, Canada; Antimicrobials Group, Murdoch Children's Research Institute, Parkville, Victoria, Australia; Department of Paediatrics, University of Melbourne, Parkville, Victoria, Australia; Department of General Medicine and Infectious Diseases, Royal Children's Hospital Melbourne, Parkville, Victoria, Australia; Infectious Disease Service, Nelson Bays Primary Health, Nelson, New Zealand; Department of Pharmaceutical Sciences, University of Colorado Skaggs School of Pharmacy and Pharmaceutical Sciences, Aurora, Colorado, USA

**Keywords:** cephalexin, Enterobacterales, methicillin-susceptible *Staphylococcus aureus*, oral transition therapy, pharmacokinetics and pharmacodynamics

## Abstract

**Background:**

Cephalexin is increasingly used as initial and oral transition therapy for severe infections, but optimal dosing in this setting remains uncertain.

**Methods:**

Four cephalexin pharmacokinetic (PK) datasets spanning preterm neonates through young adults were pooled to develop a unified population PK model. Simulations estimated probability of target attainment (PTA) and cumulative fractional response (CFR, target attainment weighted across the pathogen MIC distribution) across *f*T > MIC targets (free drug time above the minimum inhibitory concentration), MIC distributions for methicillin-susceptible *Staphylococcus aureus* (MSSA) and Enterobacterales, and multiple dosing regimens.

**Results:**

Seventy-one subjects (age 9 days–29 years) contributed 360 plasma samples, best described by a 1-compartment model with first-order absorption and lag time. Apparent volume and clearance scaled with fat-free mass, and clearance increased with postmenstrual age. Absorption was slower in neonates. Cephalexin 25 mg/kg/dose, maximum 1000 mg, every 8 hours, achieved 1-log kill targets for MSSA (PTA ≥ 98% for 35% *f*T > MIC at MIC = 4 mg/L); every 6-hour dosing was needed at MIC = 8 mg/L. For Enterobacterales, a 60% *f*T > MIC target was achieved only in infants < 2 months. However, higher-dose regimens (37.5–50 mg/kg, maximum 1500 mg, every 6 hours) achieved CFRs > 90% for 40%–50% *f*T > MIC targets when cefazolin MICs were ≤2 mg/L.

**Conclusions:**

Cephalexin PK varies with age but is well-captured by a unified size- and maturation-scaled model. These data support dosing within the FDA-approved range for systemic MSSA infections. Higher-dose regimens are necessary for adequate target attainment against Enterobacterales. Appropriately dosed cephalexin represents a viable option for selected severe infections, including oral transition therapy.

## Teaser

This pharmacokinetic analysis spanning infancy through young adulthood supports cephalexin as an oral option for selected severe infections. Standard dosing achieved pharmacodynamic targets for methicillin-susceptible Staphylococcus aureus, while higher doses were needed to improve target attainment against Enterobacterales.

Cephalexin, an oral first-generation cephalosporin, is increasingly used to treat systemic infections in both children and adults, either as initial therapy in clinically stable patients or as oral transition therapy following intravenous (IV) treatment. This reflects cephalexin's favorable pharmacologic profile—high oral bioavailability (80%–90%), low protein binding, predominantly renal elimination resulting in high urinary concentrations, good safety and tolerability across a wide dosing range, and low cost [1]—as well as its reliable activity against common pathogens, including methicillin-susceptible *Staphylococcus aureus* (MSSA), some Enterobacterales (eg, *Escherichia coli*), β-hemolytic streptococci, and *Kingella kingae*.

Despite widespread use, optimal cephalexin dosing remains uncertain. FDA-approved dosing is 25–50 mg/kg/day in equally divided doses for pediatric patients > 1 year and 250 mg q6 hours for patients ≥ 15 years, with dosing up to 100 mg/kg/day (or 4000 mg/day) for more severe infections [2]. However, substantial pediatric pharmacokinetic (PK), safety, tolerability, and efficacy data support higher dosing. Regimens of 30–50 mg/kg/dose (up to 1500 mg/dose) and total daily doses up to 150 mg/kg/day (or 6000 mg/day) appear in multiple institutional clinical pathways [3–8] and national/international treatment guidelines [9, 10]. Multiple PK studies (including Studies 2/3 in this analysis) support these higher doses [11–15], and seminal randomized pediatric osteomyelitis trials demonstrate safety and efficacy with 150 mg/kg/day divided 4 times daily [16, 17]. Consistent with these findings, a recent study of prolonged high-dose cephalexin (45 mg/kg/dose, maximum 1500 mg, 3 times daily) reported good clinical outcomes, with low rates of gastrointestinal symptoms (2.6%) and infrequent significant neutropenia [18].

There are also no universally accepted susceptibility breakpoints for cephalexin across agencies such as Clinical and Laboratory Standards Institute (CLSI), the European Committee on Antimicrobial Susceptibility Testing (EUCAST), and the US Committee on Antimicrobial Susceptibility Testing (USCAST). For severe infections, these agencies either provide no recommendations or advise against cephalexin use. For MSSA, EUCAST and USCAST recommendations are limited to non-severe, uncomplicated infections [19, 20]. For Enterobacterales, interpretive criteria are restricted to uncomplicated urinary tract infections (UTIs). This lack of clear guidance contributes to clinical practice favoring other oral options for systemic infections. However, cephalexin is increasingly used for severe infections, including osteomyelitis [10], septic arthritis [21], pyelonephritis [22–26], and bacteremia [27–34], despite the absence of established breakpoints for these indications.

This disconnect between formal breakpoints and clinical practice highlights the need for more PK and pharmacodynamic (PD) data that reflect real-world dosing and, more broadly, an improved understanding of cephalexin's PK to ensure that current dosing strategies achieve therapeutic exposures, particularly for systemic infections that may require higher doses.

To address this gap, we aimed to characterize age-related changes in cephalexin disposition across the age spectrum and evaluate the PK/PD performance of cephalexin dosing regimens both within and above the FDA-labeled doses.

## MATERIALS AND METHODS

### Study Design

This pooled population PK (popPK) analysis combined 4 cephalexin plasma concentration datasets (Table 1).

- **Study 1** [35] (NCT04916951): young infants (9–56 days) at Children's Hospital Colorado (CHCO) received a single cephalexin study dose or were receiving cephalexin clinically; weight > 2 kg, postnatal age (PNA) 9–56 days, and gestational age (GA) 29–41 weeks (12/33 [36%] preterm, <37 weeks).
- **Study 2** [12] (NCT03802552): children (9 months–17 years) hospitalized at CHCO with musculoskeletal infections received single-dose (50 mg/kg, max 1375 mg) cephalexin; only cephalexin data from this cephalexin/cefadroxil crossover study were included.
- **Study 3** [11]: non-infant children (1–16 years) with musculoskeletal infections at CHU Sainte-Justine (Montreal) sampled after an observed in-clinic steady-state cephalexin dose; typical standard-of-care dosing was 50 mg/kg/dose (max 1500 mg).
- **Study 4** [36]: healthy young adults (18–29 years old) in New Zealand who underwent rich sampling after a single 1000 mg oral dose following a standardized meal; only data without probenecid were included.

**Table 1.** Study Subject Demographics

Characteristic	Study #1: CHCO Young Infants (n = 33)	Study #2: CHCO Non-infant Children (n = 15)	Study #3: Montreal Non-infant Children (n = 12)	Study #4: NZ Healthy Young Adults (n = 11)	Combined Total (n = 71)
Sex, male (%)	23 (70%)	9 (60%)	7 (58%)	4 (36%)	43 (61%)
Age, median (range)	31.2 d (9.5–56.5)	12.22 yr (0.82–17.78)	7.61 yr (1.16–16.66)	20.42 yr (18.25–29.2)	1.46 yr (0.03–29.2)
Gestational age at birth (weeks), median (range)	37 and 2/7 (29 and 3/7–40 and 6/7)	N/A	N/A	N/A	N/A
Height (cm), median (range)	50 (42.5–60.2)	152 (66–186.5)	122.88 (88.19–182.17)	176 (162–189)	88.19 (42.5–189)
Weight (kg), median (range)	3.35 (2.20–5.39)	51.8 (7.15–79.5)	25 (10–78.8)	75 (60–112)	10.95 (2.2–112)
FFM (kg), median (range)	2.99 (1.99–4.17)	36.46 (5.27–62.18)	21.06 (7.53–63.7)	48.97 (38.74–77.18)	8.06 (1.99–77.18)
Creatinine (mg/dL), median (range)	0.27 (0.15–0.42)	0.47 (0.19–0.87)	0.39 (0.24–0.72)	0.92 (0.72–1.28)	0.34 (0.15–1.28)
eGFR (mL/min/1.73 m^2^), median (range)	68.85 (37.80–126.14)	139.39 (80.80–187.91)	126.75 (77.73–171.94)	95.54 (77.06–120.34)	101.34 (37.80–187.91)
Dose (mg/kg), median (range)	24.67 (11.97–29.46)^a^	24.13 (17.3–50.36)	40.41 (19.04–51.18)	13.33 (8.93–16.67)	24.6 (8.93–51.18)
Dose (mg), median (range)	85 (28.5–135)	1250 (360–1375)	1000 (500–1500)	1000 (1000–1000)	550 (28.5–1500)
Number of samples analyzed	144	53	53	110	360
Number of analyzed samples that were BLQ	0	0	7	15	22
Mean samples per subject	4.4	3.6	4.4	10	5.1
Number of dosing intervals in which sampling occurred					
1	25	15	3	11	54
2	4	0	9	0	13
≥3	4	0	0	0	4
Feeding status					
Meal/feed within 1 h of dose	22	9	0	11	42
≥1 h since meal/feed	10	4	0	0	14
Unknown or variable	1	2	12	0	15
Formulation					
Suspension	33	10	4^b^	0	47
Capsule	0	5	4^b^	11	20
Unknown	0	0	4	0	4

Abbreviations: CHCO, Children's Hospital Colorado; NZ, New Zealand; FFM, fat-free mass; eGFR, estimated glomerular filtration rate; BLQ, below the limit of quantification.

^a^One subject received 12.0 mg/kg/dose, while all other subjects received 22.5–29.5 mg/kg/dose.

^b^Formulation imputed for subjects in Study #3. See Supplementary Appendix A.

All studies had institutional review board approval, and written informed consent was obtained prior to study procedures. Additional details on inclusion/exclusion criteria, dosing and sampling, and study protocols are available in the respective publications. Across all studies, cephalexin dosing, PK sampling, total body weight (TBW), and sex were available. Missing values for height, serum creatinine, GA, formulation, and feeding status (ie, co-administration with a meal) were either imputed or excluded (Supplementary Appendix A).

### Model Development

A popPK model was developed using non-linear mixed-effects modeling in MonolixSuite2024R1 (Lixoft SAS, Antony, France). Multiple absorption, compartmental, and residual error structures were tested. Covariates were assessed using stepwise forward addition (*P* < .05) and backward elimination (*P* < .01) to identify statistically and biologically meaningful relationships with TBW, fat-free mass (FFM), postnatal age (PNA), postmenstrual age (PMA), creatinine, estimated glomerular filtration rate (eGFR), formulation, and feeding status. Supplementary Appendix A includes calculations used for FFM and eGFR. Model adequacy was evaluated using parameter plausibility and precision, objective function values, diagnostic plots, shrinkage, and non-parametric bootstrap. Further details on model development, including methods for below the limit of quantification (BLQ) handling and data exclusion, are in Supplementary Appendices B–D.

### Outcomes

Simulations were performed in Simulx 2024R1 (Lixoft SAS) to estimate PK parameters for virtual subjects and evaluate area under the concentration-time curve (AUC) and probability of target attainment (PTA) for various cephalexin doses and PD targets. Virtual populations—generated in PK-Sim (Open Systems Pharmacology, https://www.open-systems-pharmacology.org/) using NHANES and neonatal distributions for weight, height, PNA, and GA—consisted of 11 subgroups of 5000 subjects each: 4 young infant groups stratified by GA (30–34 weeks vs ≥35 weeks) and PNA (7–28 days vs 29–60 days), excluding neonates < 7 days (not represented in model development), as well as populations for 2−<6 months, 6−<12 months, 1−<4 years, 4−<7 years, 7−<12 years, 12−<18 years, and 18–30 years.

For each virtual subject, 24-hour steady-state AUC (AUC_0–24,SS_) was calculated as dose (mg) divided by individual estimated oral clearance (L/hour), multiplied by the number of daily doses. Pediatric regimens were optimized to match adult reference AUC_0–24,SS_ values for 1000 mg q6 hours (Supplementary Table 1).

MSSA and Enterobacterales were selected as representative pathogens for high-exposure cephalexin use, as other relevant organisms (eg, group A/C/G streptococci, *K. kingae*) typically exhibit similar or lower cephalexin MICs.

PTA was evaluated across various PD targets, MICs, and dosing regimens (Supplementary Appendix E). Because no clinical studies have established optimal PD targets for oral β-lactams, primary targets were based on a neutropenic murine thigh PD model for cephalexin [37, 38]. Primary targets were 35% *f*T > MIC for MSSA (reflecting 1-log kill) and 60% *f*T > MIC for Enterobacterales (reflecting bacterial stasis). Given uncertainty in the optimal PD target, a broader range (20%–100% *f*T > MIC) was evaluated in exploratory analyses, encompassing the ∼25% *f*T > MIC stasis target for MSSA and 40%–60% *f*T > MIC targets associated with bacterial stasis and killing across the cephalosporin class [37–41]. The evaluated MIC range (1–16 mg/L) reflects clinically relevant distributions for MSSA and Enterobacterales. While some wild-type Enterobacterales have cephalexin MICs > 16 (tentative ECOFF 32 mg/L per EUCAST), nearly all isolates with cefazolin MICs ≤ 2 mg/L have corresponding cephalexin MICs ≤ 8–16 mg/L [42].

The evaluated dosing range spans both FDA-approved regimens (up to 25 mg/kg/dose, max 1000 mg) and higher doses (up to 50 mg/kg/dose, max 1500 mg). For children < 12 years, 7 dosing regimens were evaluated: 16.6 mg/kg (max 500 mg) q8 hours, 25 mg/kg (max 1000 mg) q6 or q8 hours, 37.5 mg/kg (max 1500 mg) q6 or q8 hours, and 50 mg/kg (max 1500 mg) q6 or q8 hours, with the fixed maximum doses used for adolescents (12–18 years) and young adults.

Population-level performance was assessed using cumulative fractional response (CFR), calculated as a weighted average of the PTAs for the fraction of isolates at each discrete MIC, across the MIC distribution of interest. MIC distribution data are outlined in Supplementary Appendix E. For MSSA, CFR was evaluated using 2 wild-type MIC distributions to account for potential geographic variability: our local distribution (MIC_90_ = 4 mg/L) and the USCAST distribution, which included a greater proportion of isolates with higher MICs (MIC_90_ = 8 mg/L) [37, 43]. Enterobacterales CFRs reflect CLSI-reported isolates with cefazolin MIC ≤ 2 mg/L (for which cephalexin MICs are 4–16 mg/L) [42].

PTA and CFR were calculated over the 24–48-hour interval after the first dose to reflect steady-state exposures, with >90% considered optimal. Protein binding was fixed at 10% across all populations [2].

## RESULTS

### Study Population

The analysis included 360 plasma concentrations obtained from 71 subjects receiving enteral cephalexin across 4 studies (see Supplementary Appendix D for excluded data). This included 33 infants < 60 days, 27 older children (9 months–17 years), and 11 young adults (18–29 years). No samples were BLQ in Studies 1 and 2, while 7 samples were BLQ (≤0.625 mg/L) in Study 3 and 15 (≤0.2 mg/L) in Study 4, representing 6% of analyzed samples. Subject characteristics are summarized in Table 1.

### Final Model

A 1-compartment model with first-order absorption and lag time best described the data. A combined residual error model was used, with inter-individual variability (IIV) supported for the lag time (Tlag), first-order rate constant (Ka), and apparent clearance (CL/F; F, bioavailability) but not apparent volume of distribution (Vd/F) due to high shrinkage (>50%). Body size, represented by FFM, was a major determinant of variability. CL/F maturation was included as a Hill function using PMA. PNA influenced Ka in neonates, incorporated as a fixed scalar only for subjects ≤ 21 days old. No other covariates were retained. BLQ data were handled using Monolix's censored-likelihood (Beal M4) method. The final model demonstrated acceptable precision in parameter estimates (Table 2). See Supplementary Appendices B and C for model development and final model details.

**Table 2.** Final Cephalexin Population Pharmacokinetic Model Parameters

Parameter	Final Model Estimate	RSE%	Bootstrap (N = 1000) Median (95% CI)
Fixed effects			
Tlag (h)	0.53	13.16	0.49 (0.32–0.63)
Ka (1/h)	1.59	18.2	1.51 (0.78–2.16)
β_age_ _<_ _21d_Ka_	−0.81	34.23	−0.82 (−1.56 to −0.25)
Vd/F (L/56 kg)	22.04	7.57	22.06 (11.16–26.26)
β_FFM_V_	0.92	2.7	0.91 (0.79–0.97)
CL/F (L/h/56 kg)	16.6	4.25	16.33 (14.89–17.87)
β_FFM_CL_	0.82	2.82	0.81 (0.71–0.91)
Hill coefficient	5.06	15.51	5.49 (3.44–7.99)
TM_50_ (weeks PMA)	42.96	2.08	43.14 (39.07–49.81)
Random effects (CV%)			
IIV for Tlag (CV%)	87.13%	15.18	0.77 (0.51–1.11)
IIV for Ka (CV%)	51.87%	23.58	0.45 (0.18–0.7)
IIV for CL/F (CV%)	17.25%	14.98	0.16 (0.097–0.23)
Error model parameters			
Constant (a, mg/L)	0.31	18.01	0.26 (0.06–0.42)
Proportional (b)	0.21	9.39	0.21 (0.16–0.28)

Abbreviations: RSE, relative standard error; Tlag, lag time (hours); Ka, absorption rate constant (1/h); β, covariate coefficient; F, bioavailability; Vd/F, apparent volume of distribution (in liters, normalized to 56 kg); CL/F, apparent clearance (in liters per hour, normalized to 56 kg); FFM, fat-free mass (kg); PMA, postmenstrual age; TM_50_, PMA at which 50% of the maximum value is reached; IIV, inter-individual variability; CV, coefficient of variation.

The visual predictive check (Figure 1) showed that the model captured the observed data appropriately, with 89.7% (323 of 360) observations falling within the 90% prediction interval generated by the simulations.

**Figure 1.**
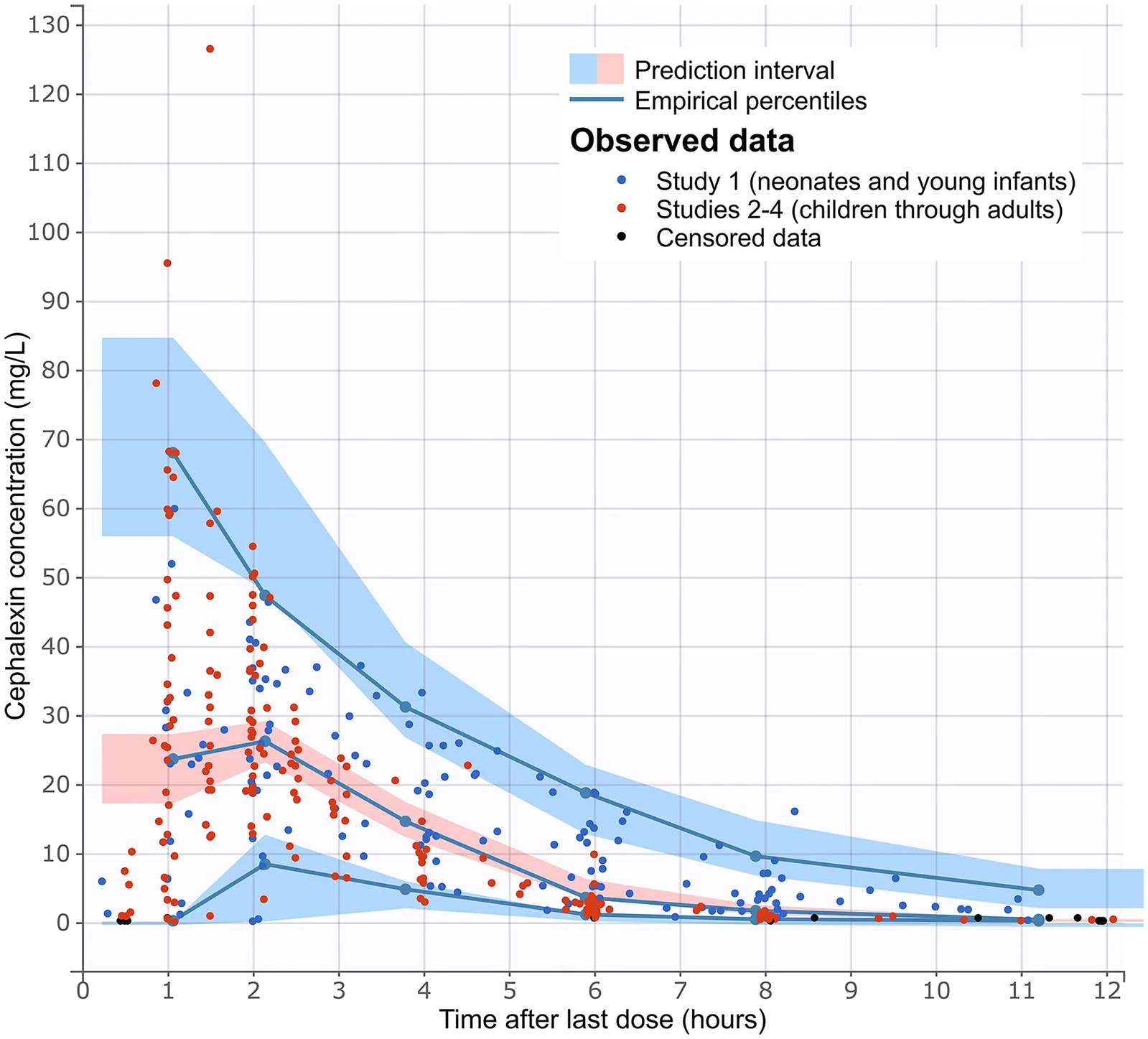
Visual predictive check (without prediction correction) for the final pharmacokinetic model. Blue data points reflect neonatal data from Study 1. Red data points reflect data from children and young adults (Studies 2–4). Black data points reflect censored/below-limit-of-quantification (BLQ) data. Solid lines represent the 5th, 50th, and 95th percentiles of the observed data, while shaded areas show the 95% prediction intervals around these 5th, 50th, and 95th percentiles for the simulated data. One BLQ sample collected at 15.33 h after last dose was truncated from the plot for display purposes.

The supplement includes individual fits (Supplementary Figure 1), individual empirical Bayesian estimates (Supplementary Table 2), and comparisons of Ka versus PNA (Supplementary Figure 2) and weight-normalized CL versus PMA (Supplementary Figure 3) and PNA (Supplementary Figure 4). CL increased from a median 0.54 L/hour in neonates ≤ 28 days to 14.8 L/hour in young adults. Weight-normalized CL peaked in early childhood (∼1–5 years) and was lowest during the early neonatal period. Neonates ≤ 28 days also had the longest Tlag, slowest Ka, and highest Vd per kg TBW.

### AUC Matching

For a 1000 mg dose, the virtual young adult population had a median AUC_0-inf_ of 65.9 mg·h/L (SD 17.3) and an AUC_0–24,SS_ of 263.7 mg·h/L with q6-hour dosing. This matched published data: mean AUC_0-inf_ of 68.1 (AUC_0–24,SS_ 272.4) from the Study 4 NCA analysis and mean AUC_0-inf_ of 67.46 (AUC_0–24,SS_ 269.8) from 13 adult cephalexin AUC values across formulations (capsule, tablet, suspension) and fed/fasting status (Supplementary Table 1).

Supplementary Figure 5 shows simulated AUCs by subgroup using AUC-matched doses: 25 mg/kg (max 1000 mg) q6 hours (12–<18 years), q8 hours (2 months–<12 years), or q12 hours (<2 months). This dosing produced an AUC_0–24,SS_ within 15% of the adult reference, except in premature neonates (AUC_0–24,SS_ 51% higher for neonates ≤ 28 days with GA < 35 weeks). The apparent lower dosing requirement in children 2 months–<12 years was an artifact of the 1000 mg dose cap, which resulted in lower weight-based doses in adolescents and young adults.

### Probability of Target Attainment Analysis

#### MSSA

Cephalexin 25 mg/kg/dose (max 1000 mg) q8 hours achieved ≥98% PTA for the 1-log kill target (35% *f*T > MIC) at MIC = 4 mg/L across all age groups (Figure 2; Supplementary Table 3), with >99% CFR for the CHCO MIC distribution (MIC_90_ = 4 mg/L) [37, 43]. For the higher MIC distribution (MIC_90_ = 8 mg/L; Supplementary Appendix D), this regimen achieved >90% CFR for bacterial stasis (25% *f*T > MIC), but achieving ≥90% CFR for 1-log kill required either more frequent dosing (25 mg/kg [max 1000 mg] q6 hours) or a higher q8-hour dose (37.5 mg/kg [max 1500 mg] q8 hours; Figures 3*A*; Supplementary Figure 6).

**Figure 2.**
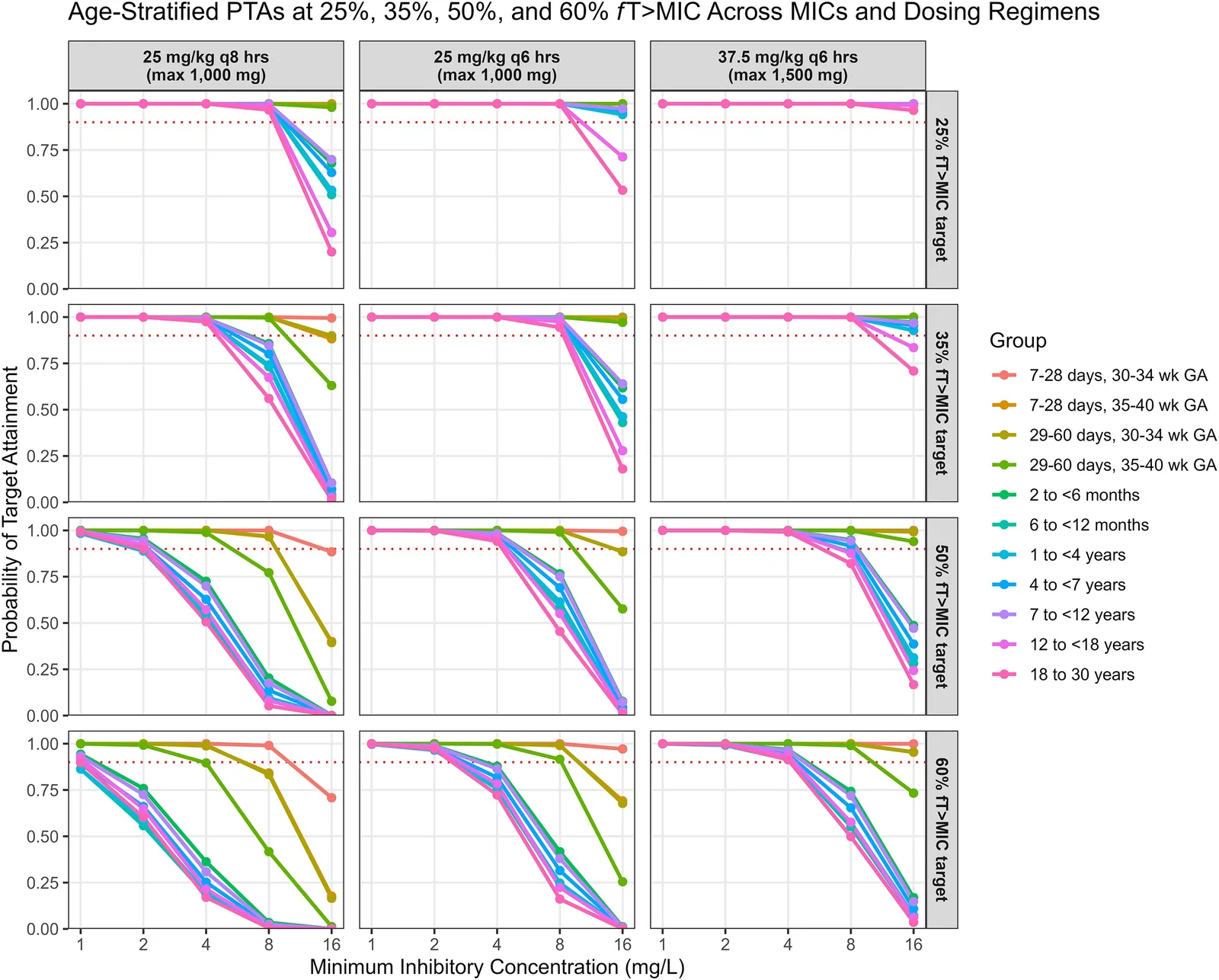
Probability of target attainment (PTA) for age groups across minimum inhibitory concentrations (MICs) 1–16 mg/L for 3 example dosing regimens and 4 PD targets (25%, 35%, 50%, and 60% *f*T > MIC). The 25% and 35% targets correspond to bacterial stasis and 1-log kill against methicillin-susceptible *Staphylococcus aureus*, respectively; 50% represents an intermediate cephalosporin PD target, and 60% corresponds to the Enterobacterales stasis target. The red dotted line represents 90% PTA.

**Figure 3.**
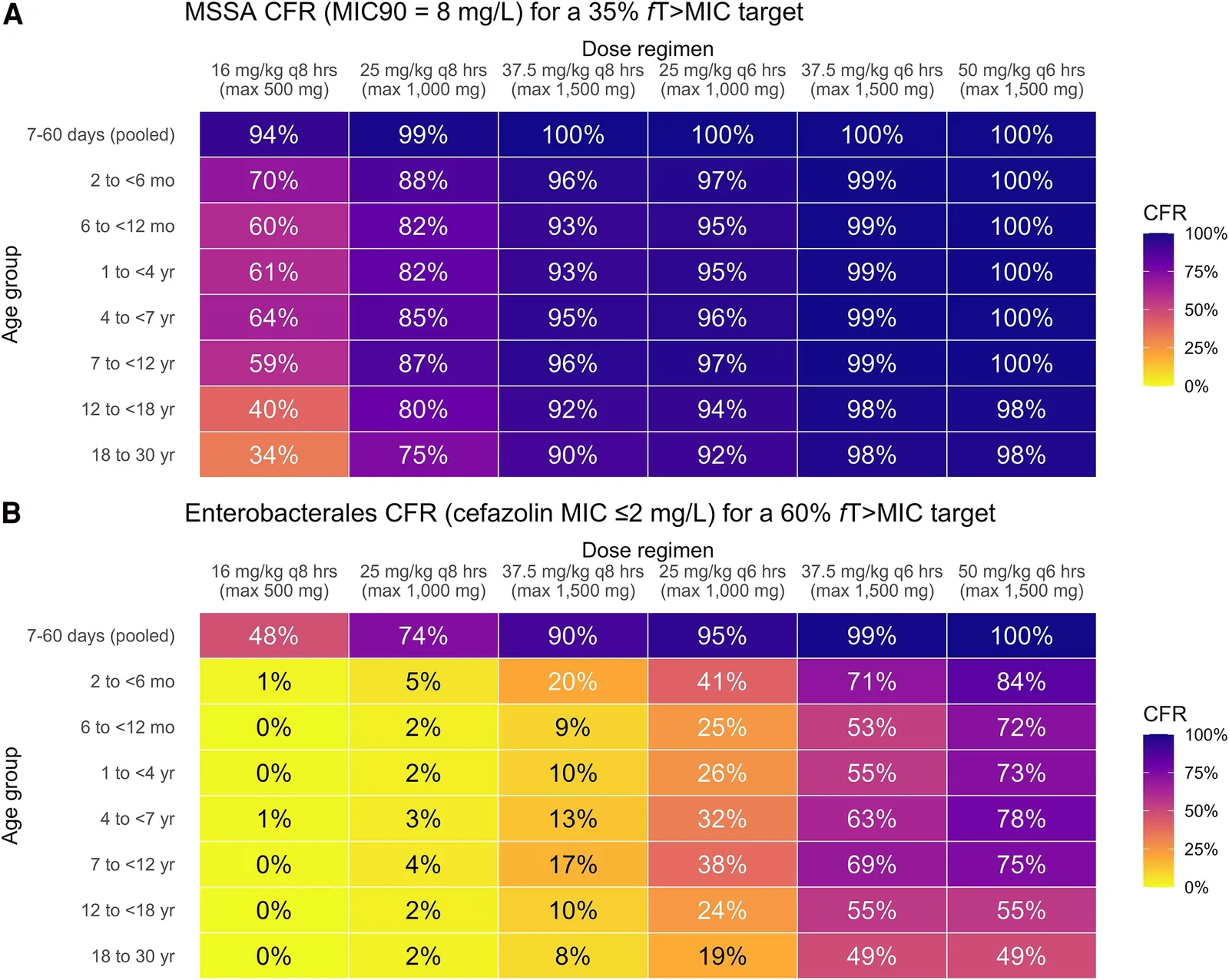
Heatmap of cumulative fractional response (CFR) values across age groups and dose regimens. Rows represent age strata and columns represent cephalexin dosing strategies. Tile shading corresponds to the proportion of simulated patients achieving the CFR target for the given MIC distribution. (A, top) CFRs against methicillin-susceptible *Staphylococcus aureus* (MSSA) for a 35% *f*T > MIC target, using the USCAST MSSA MIC distribution (MIC_90_ = 8 mg/L). (B, bottom) CFRs against Enterobacterales for a 60% *f*T > MIC target, using the CLSI Enterobacterales MIC distribution for isolates with cefazolin MICs ≤ 2 mg/L (corresponding to cephalexin MICs of 4–16 mg/L).

Higher dosing further increased target attainment: 37.5 mg/kg (max 1500 mg) q6 hours achieved ≥90% CFR at 65% *f*T > MIC for MIC_90_ = 4 mg/L and at 50% *f*T > MIC for MIC_90_ = 8 mg/L (Supplementary Figure 6). This corresponds to the exposure range in the murine model where bacterial killing plateaus (≥40%–50% *f*T > MIC), approximately 2-log kill [37].

#### Enterobacterales

In contrast, cephalexin did not reliably achieve the more stringent Enterobacterales target (60% *f*T > MIC) [37], with meaningful target attainment at MICs 8–16 mg/L limited to young infants (Figure 3*B*).

In infants ≤ 2 months, PTAs > 90% at MIC = 16 mg/L were achieved with high-dose regimens in neonates ≤ 28 days (37.5 mg/kg q6 hours) and infants 29–60 days (50 mg/kg q6 hours) (Supplementary Table 3). Beyond early infancy, no evaluated regimen achieved >90% PTA at MICs of 8–16 mg/L. In children 2 months–<18 years, even the highest evaluated regimen (50 mg/kg q6 hours) yielded CFRs of only 55%–84% (Supplementary Figure 7). Similarly, in young adults, cephalexin 1000 mg q6 hours resulted in poor target attainment (PTA 16% at MIC = 8 mg/L; <1% at 16 mg/L; CFR 18.6% [Supplementary Table 3]).

Given uncertainty in the optimal PD target, we also evaluated which *f*T > MIC thresholds are achievable across dosing regimens. Lower targets were consistently achievable using 25 mg/kg (max 1000 mg) q6 hours, with >90% CFRs observed at 30% *f*T > MIC in adults, 35%–40% in children, and 55%–100% in young infants. Increasing the dose to 37.5 mg/kg (max 1500 mg) q6 hours extended achievable targets to ≥40%–50% *f*T > MIC (Supplementary Figure 7, Supplementary Table 4). Table 3 presents age-specific dosing strategies designed to achieve 40%, 50%, or 60% *f*T > MIC targets against Enterobacterales.

**Table 3.** Cephalexin Dosing Recommendations for Treatment of Enterobacterales Infections to Achieve Either >90% Probability of Target Attainment for MICs ≤ 16 mg/L or >90% Cumulative Fractional Response for Isolates With Cefazolin MICs ≤ 2 mg/L Across Multiple *f*T > MIC Targets

	For >90% PTA for MICs up to 16 mg/L		For >90% CFR When Isolates Have Cefazolin MICs ≤ 2		
Age	60% *f*T > MIC	40% *f*T > MIC	60% *f*T > MIC	50% *f*T > MIC	40% *f*T > MIC
7–28 d	37.5 mg/kg every 6h	25 mg/kg every 6h	25 mg/kg every 6h	25 mg/kg every 8h	25 mg/kg every 8h
29–60 d	50 mg/kg every 6h	25 mg/kg every 6h	37.5 mg/kg every 6h	25 mg/kg every 6h	25 mg/kg every 8h
2 mo–7 yrs	n/a	50 mg/kg every 6h	n/a	50 mg/kg every 6h	37.5 mg/kg every 6h
7–12 yrs	n/a	50 mg/kg every 6h	n/a	37.5 mg/kg every 6h	25 mg/kg every 6h
12–18 yrs	n/a	n/a	n/a	n/a	1500 mg every 6h
18–30 yrs	n/a	n/a	n/a	n/a	1500 mg every 6h

Abbreviations: PTA, probability of target attainment; CFR, cumulative fractional response; MIC, minimum inhibitory concentration; n/a, no evaluated dosing regimen (within the studied dose range) achieved this target.

## DISCUSSION

This study provides a unified, age-spanning characterization of cephalexin PK and links drug exposure to PD target attainment across clinically relevant dosing regimens. Using pooled data from preterm neonates through young adults, we identified age-dependent differences in exposure driven primarily by maturation of clearance. These results have important dosing implications: high-exposure regimens capable of achieving PD targets for MSSA (35% *f*T > MIC) were readily attainable, while achieving stringent Enterobacterales targets (60% *f*T > MIC) was rarely feasible, even with aggressive dosing.

Our PK findings are consistent with previously published cephalexin data. Prior studies have shown slower absorption in neonates < 3 days old [13] and in infants and young children [14], without an apparent age effect afterwards, which aligns with our model. Clearance estimates were consistent with previously reported values for the individual datasets included in this model: Study 1 (0.67 L/hour vs 0.66 L/hour [35]), Study 2 (11.8 L/hour vs 10.8 L/hour [12]), Study 3 (0.263 L/hour/kg vs 0.289 L/hour/kg [15]), and Study 4 (14.8 L/hour vs 14.7 L/hour [36]). Adult clearance estimates also fell within published ranges (12–18 L/hour, ∼200–300 mL/min) [44], and volume of distribution estimates were comparable.

Simulated AUCs were similarly concordant with published data (Supplementary Table 1). Consistent with known developmental pharmacology, our exposure-matching analyses showed that children require higher weight-based doses than adults to achieve comparable exposures due to increased clearance. However, because adult dosing is constrained by fixed maximum doses, children typically achieve higher overall exposures with standard mg/kg regimens.

### MSSA

Our simulations indicate that cephalexin dosing within the FDA-approved range is sufficient for high target attainment against MSSA, supporting the doses recommended in the PIDS/IDSA [10] and ESPID [9] osteomyelitis guidelines. Either 25 mg/kg (max 1000 mg) q6 hours or 37.5 mg/kg (max 1500 mg) q8 hours achieves 1-log kill for MICs ≤ 8 mg/L, while lower q8-hour dosing is sufficient for lower MICs (≤4 mg/L) or for a bacterial stasis target. This is consistent with the high clinical success observed when these FDA-labeled and guideline-concordant doses are used for MSSA osteomyelitis and bacteremia in children [45–48].

These findings support the USCAST breakpoint of 8 mg/L for *S. aureus* [20], yet both USCAST and EUCAST advise against using cephalexin for severe MSSA infections. Our results challenge this limitation. Cephalexin reliably achieves 1-log kill, not solely stasis, at clinically relevant exposures, and higher dosing—37.5 mg/kg (max 1500 mg) q6 hours—approaches 2-log killing.

### Enterobacterales

While PK and clinical data clearly support cephalexin's use for systemic MSSA infections, its role and optimal dosing for Enterobacterales infections are less certain. Our simulations show poor target attainment using Enterobacterales’ higher PD target (60% *f*T > MIC) and less favorable MICs, supporting CLSI, EUCAST, and USCAST's recommendation against using cephalexin as initial therapy for severe Enterobacterales infections.

The optimal PD target for Enterobacterales, however, remains unknown. Cephalosporin class data suggest bacteriostasis occurs at ∼30%–40% *f*T > MIC with near-maximal effects at ∼60%–70% [38–41], but cephalexin-specific data are limited to a murine thigh model of 5 *E. coli* isolates in which stasis occurred from 34% up to >61% *f*T > MIC. On this basis, USCAST adopted ∼61% *f*T > MIC as a stasis target [37].

However, applying this stringent target appears discordant with clinical outcomes. For pyelonephritis, typical pediatric dosing (25 mg/kg q6–8 hours) produced CFRs < 50% in our simulations, yet clinical cure rates remain high [22–26]. In the pediatric SCOUT UTI trial, cephalexin 16.6 mg/kg q8 hours achieved 97.9% cure in febrile UTI despite simulated CFRs < 1% [23]. This discrepancy may partly reflect urinary physiology, where active tubular secretion produces urinary concentrations far exceeding plasma, and bacterial burdens in the kidney are lower (and intermittently flushed with voiding) than in experimental models. However, it may also indicate that the 60% *f*T > MIC target overestimates the exposure required for clinical success in UTIs.

Similarly, oral transition therapy for Enterobacterales bloodstream infections has demonstrated clinical success despite cephalexin dosing with low predicted target attainment. Several adult studies have compared oral β-lactams (including cephalexin) with alternatives such as trimethoprim-sulfamethoxazole or fluoroquinolones. While some have reported higher recurrence rates with oral β-lactams, most patients in those studies still achieved clinical cure despite low cephalexin dosing (eg, ∼500 mg q6–12 hours) [33, 34]. More recent analyses have found similar outcomes using β-lactams, even when dosing remained suboptimal [27–32]. Our simulations suggest that substantially improving PD target attainment would require both higher and more frequent cephalexin doses: increasing adult dosing from 1000 mg q8 hours to 1500 mg q6 hours increased Enterobacterales CFRs from 1.6% to 48.6% using a 60% *f*T > MIC target and to 94% using a 40% target.

### Initial Versus Oral Transition Therapy

The most common use of cephalexin for severe infections is as oral transition following initial IV therapy. However, the PD targets used in our simulations were derived from murine models of initial therapy. This may overestimate exposures needed for oral transition: bacterial burden is substantially reduced following initial IV therapy, MICs may decrease at lower inocula [49], and *in vitro* data suggest oral therapy is more effective against the lower bacterial inocula typical of transition therapy than against the higher inocula present with initial treatment [50]. For MSSA, favorable clinical outcomes with oral transition suggest that the 1-log kill target may exceed the exposure needed after initial IV therapy. Similarly, for Enterobacterales, clinical success despite poor predicted target attainment suggests that the 60% stasis target may be overly stringent for pyelonephritis and oral transition of other systemic infections. Nevertheless, in the absence of validated clinical PD targets for oral therapy, we used these cephalexin-specific experimental targets as the best available PD benchmarks.

### Dosing Recommendations

Our simulations and available clinical data support a context-specific approach to cephalexin dosing based on pathogen and phase of therapy.

For initial treatment of severe MSSA infections, we favor either 25 mg/kg (max 1000 mg) q6 hours or 37.5 mg/kg (max 1500 mg) q8 hours, both of which achieve 1-log kill for MICs ≤ 8 mg/L. For oral transition, 25 mg/kg (max 1000 mg) q8 hours is likely sufficient as it achieves bacterial stasis across the evaluated MIC distributions and 1-log kill for MICs ≤ 4 mg/L. For higher organism burden, limited IV lead-in, or concern for decreased infection-site drug penetration, higher q8-hour doses or q6-hour dosing may provide a greater margin of safety.

For Enterobacterales, our data do not support cephalexin as initial therapy for severe infections. Pediatric pyelonephritis is an important exception; we favor 25 mg/kg (max 1000 mg) q8 hours based on clinical experience and outcome data, which achieves >90% CFR when PD targets are set near the lower end of the cephalosporin range (∼30%–40% *f*T > MIC). For oral transition of other systemic Enterobacterales infections, we consider ∼40%–50% *f*T > MIC a pragmatic target pending better clinical PD data. Higher-dose regimens (37.5–50 mg/kg, max 1500 mg, q6 hours) achieve acceptable CFRs at these targets when cefazolin MICs are ≤2 mg/L and remain within studied PK and safety ranges.

Finally, dosing frequency should balance PK/PD exposure with practicality and adherence. When target attainment is comparable, we favor q8-hour dosing, though q6-hour dosing is appropriate when the additional exposure is needed.

### Limitations

This study has several limitations. The PK dataset combined studies spanning a wide age range with differing doses, populations, sampling intensity, and analytical methods; thus, some variability may reflect study-design differences rather than biological variation. Sparse absorption-phase sampling limited assessment of absorption models, though the impact on target attainment is likely modest because clearance is the primary determinant of *f*T > MIC. PD targets were derived from murine models rather than human clinical data, which may underestimate clinical success. Plasma-based simulations may not fully reflect bone exposure but remain reasonable, as cephalexin achieves measurable bone and synovial fluid concentrations and bone penetration has not been definitively linked to clinical outcomes [51]. Simulations used literature-derived protein binding estimates, which may vary with age or physiologic state, and albumin data were not available. The simulated populations also reflect body weights typical of the underlying studies, limiting extrapolation to patients with obesity; similarly, adult data were limited to participants < 30 years. In some settings, co-administration of probenecid is used to inhibit renal tubular secretion and prolong cephalexin exposure as an alternative to dose escalation; however, this approach was not evaluated in this study. Finally, conclusions about clinical effectiveness—particularly for Enterobacterales infections beyond pyelonephritis—are limited by the lack of pediatric cephalexin trials. Thus, these PK/PD-based findings provide a dosing framework but should be interpreted alongside clinical evidence and applied with clinical judgment.

### Conclusions

For severe MSSA infections, cephalexin is a highly effective oral agent, with FDA-approved dosing (25 mg/kg, max 1000 mg, q6–8 hours) sufficient to achieve 1-log kill targets. In contrast, for Enterobacterales infections, standard dosing achieves low target attainment, particularly for the 60% *f*T > MIC threshold. However, clinical data suggest that this PD target may overestimate the exposure needed for clinical success. For severe Enterobacterales infections, including oral transition therapy for bloodstream infections, higher-dose regimens (37.5–50 mg/kg, max 1500 mg, q6 hours) meaningfully improve PD target attainment. These doses should be considered in future trials evaluating oral transition therapy, and CLSI and other agencies should consider the use of cefazolin surrogacy (when cefazolin MICs are ≤2 mg/L) to predict cephalexin efficacy for systemic Enterobacterales infections. Overall, appropriately dosed cephalexin is a viable treatment option across a range of severe infections, supporting a context-specific approach based on patient age, pathogen, infection site, and treatment phase.

## Supplementary Material

ofag611_Supplementary_Data

## Supplementary Data

Supplementary materials are available at *Open Forum Infectious Diseases* online. Consisting of data provided by the authors to benefit the reader, the posted materials are not copyedited and are the sole responsibility of the authors, so questions or comments should be addressed to the corresponding author.
