# Supplementary material for: Cephalexin Model-Informed Dosing Recommendations for Severe Infections: A Pooled Pharmacokinetic Analysis From Infancy to Adulthood
Source: Open Forum Infect Dis. 2026 Sep 26;13(10):ofag611. doi: 10.1093/ofid/ofag611 (PMC13678201; doi:10.1093/ofid/ofag611)
Supplement: Supplementary file 1 [file ofag611_supplementary_data.docx]

**Supplementary Information**

Cephalexin Model‑Informed Dosing Recommendations for Severe Infections: A Pooled Pharmacokinetic Analysis From Infancy to Adulthood

Haynes AS, et al.

| **Appendix A**: Imputing Missing Data and Covariate Calculations....................................... | 2-3 |  |
| --- | --- | --- |
| **Appendix B**: Model Development Workflow...................................................................... | 4-5 |  |
| **Appendix C**: Final Model Details........................................................................................ | 6-7 |  |
| **Appendix D**: Excluded Data................................................................................................ | 8 |  |
| **Appendix E**: Probability of Target Attainment and Cumulative Fractional Response........ | 9-10 |  |
| **Supplementary Tables and Figures**................................................................................... | 11-19 |  |
| **References**............................................................................................................................ | 20-21 |  |
|  |  | |

**Appendix A:** Imputing Missing Data and Covariate Calculations

*Weight/Height*: Total body weight (TBW) was available for all study subjects. Height values were available only for Studies 1, 2, and 4. Missing height values for subjects from Study 3 were imputed for use in calculations for estimated glomerular filtration rate (eGFR), lean body mass (LBM), and fat free mass (FFM). Subjects were assigned the median sex-specific BMI-for-age based on WHO reference data, and then the subject’s height was imputed (height (cm) = sqrt[weight (kg) / BMI (kg/m^2^)] × 100) assuming a median BMI for the subject’s known age and sex.

*Other Body Size Metrics*: LBM was calculated using the Peters et al. (2011)^1^ allometric equation based on TBW and height (observed or imputed). FFM was estimated using two approaches. (1) Al-Sallami et al. (2015)^2^ equation for children and adolescents, with TBW used for subjects <3 years and the Janmahasatian^3^ adult equation for subjects ≥18 years. (2) O’Hanlon et al. (2023) method,^4^ in which adult-referenced FFM was calculated from weight and height (observed or imputed) using a Janmahasatian-type equation. For subjects <20 years, this value was scaled by an age- and sex-dependent fraction of adult FFM derived from postmenstrual and postnatal age; for subjects ≥20 years, the fraction was set to 1. Final FFM was capped at 95% of body weight to avoid implausible values.

*Creatinine*: Serum creatinine was available for all subjects except for eight subjects in Study 1 and two subjects in Study 3. For Study 1 subjects, missing creatinine values were imputed using linear regression based on age from the available neonatal dataset. For Study 3 subjects, a creatinine value was imputed using exponential regression model with available creatinine data from the two pediatric datasets.

*Estimated Glomerular Filtration Rate (eGFR)*: Serum creatinine values (observed or imputed) were used to calculate an eGFR for each subject. For pediatric subjects <18 years, eGFR was calculated using the bedside Schwartz equation: eGFR (mL/min/1.73 m²) = (k × height in cm) / serum creatinine in mg/dL, with age- and sex-specific coefficients (k) of 0.31 for infants ≤28 days, 0.357 (males) or 0.331 (females) for infants >28 days, 0.413 for non-infant children <18 years.^5-7^ For adult subjects ≥18 years, eGFR was calculated using the CKD-EPI Creatinine Equation (2021, Race-Free).

*Gestational age*: Gestational age (GA) was available only for the neonatal dataset (Study 1), with a value of 40 weeks assigned to older subjects.

*Formulation*: Formulation (receipt of either capsule or suspension) was available for all studies except Study 3. Formulation was imputed for 8 of the 12 subjects. Four subjects received doses that did not correspond to an available capsule size, and they were assumed to have received suspension. Four subjects received doses rounded to an available capsule size and their mg/kg dose had reached a maximum, so they were assumed to have received capsules. The remaining subjects were assigned an unknown status.

*Feeding Status*: Feeding status prior to dosing was recorded only in Studies 1, 2, and 4, though was missing for two Study 2 subjects. All missing subjects were assigned a value of unknown for their feeding status.

*Race/Ethnicity*: Race and ethnicity were available for most subjects in Studies 1 and 2 but not collected in Studies 3 or 4. Because these variables are not known to meaningfully influence oral β‑lactam pharmacokinetics (PK) and lacked biological plausibility for affecting cephalexin disposition, they were not evaluated as covariates.

**Appendix B:** Model Development Workflow

*Overview*: The population pharmacokinetic (popPK) model was developed using MonolixSuite2024R1 (Lixoft SAS, Antony, France) following a structured, stepwise workflow. This included identification of the base structural model, selection of the residual error model, covariate assessment, and model evaluation. Parameter estimation was performed using the Stochastic Approximation Expectation-Maximization (SAEM) algorithm. Model selection was guided by reductions in the objective function value (OFV) and corrected Bayesian Information Criterion (BICc), and by assessment of parameter precision via relative standard error (RSE). An RSE <30% was considered acceptable for both fixed and random effect estimates. Graphical diagnostics, including goodness‑of‑fit (GOF) plots and visual predictive checks (VPCs), were used to evaluate agreement between observed and predicted concentrations and assess predictive performance. In addition, the proportion of observed concentrations within the 90% prediction interval was quantified. Parameter uncertainty was further assessed using the Monolix built‑in bootstrap procedure, applying a non‑parametric bootstrap with case resampling for 1,000 runs.

*Base Structural Model*: Initial structural models were selected based on PK principles and physiologic plausibility. Both one- and two-compartment structural models were compared. Multiple absorption models were explored: zero‑ and first‑order models (with and without lag time [Tlag]), transit compartment models, and a Weibull function. Residual error was evaluated using constant, proportional, and combined models.

*Handling BLQ Data*: Below‑limit‑of‑quantification (BLQ) observations were handled using Monolix’s censored‑data likelihood approach (equivalent to the Beal M4 method). Alternative strategies were also tested, including treating all BLQ values as missing, ignoring only terminal BLQ samples, and substituting half the lower-limit-of-quantification.

*Covariate Evaluation*: Body size effects on CL/F and Vd/F were evaluated using total body weight (TBW) normalized to 70 kg, as well as lean body mass (LBM) and fat‑free mass (FFM) normalized to 56 kg. These were included as part of the base model prior to the covariate search process.

Additional covariate relationships were evaluated using a stepwise approach. Forward addition was guided by an OFV reduction ≥3.84 (p < 0.05), and backward elimination used an OFV reduction ≥6.64 (p < 0.01). Covariates were included only if biologically plausible and statistically significant. The automated covariate search function in Monolix was used for this process, with only one covariate added or removed at each step. The search was started after incorporating body‑size effects on Vd/F and CL/F; however, notably, inclusion of FFM on CL/F produced the largest OFV drop of all tested covariates relative to a base model without a body size scalar.

Candidate covariates included the above body‑size metrics (TBW, LBM, FFM) as well as clinical variables. Continuous covariates included height, postnatal age (PNA), gestational age (GA), post-menstrual age (PMA), serum creatinine, and estimated glomerular filtration rate (eGFR). Categorical covariates included discretized PNA groups and feeding status categories. Continuous covariates other than weight were centered on the weighted mean and evaluated using exponential and power relationships; sigmoidal/Hill functions were also tested for time‑varying covariates such as PNA and PMA.

For PMA effects on CL/F, both fixed (Hill coefficient = 3.4; TM_50_ = 47.7 weeks) and freely estimated Hill models were evaluated. Parameteres were retained if estimated with good precision and values were physiologically plausible. For PNA effects on absorption (Ka), multiple parameterizations were explored, including: separate Ka values for neonatal versus non‑neonatal subjects; fixed scalars applied to specific PNA groups using various one‑week cutoffs; PNA modeled as a continuous or discretized covariate across the full population or specific subgroups; and sigmoidal maturation functions allowing Ka to increase with age toward a maximal value.

Additional covariate evaluation included Monolix internal statistics (Pearson correlation for continuous covariates, one-way ANOVA for categorical covariates) and visual inspection of eta vs covariate plots.

**Appendix C:** Final Model Details

*Structural model*: A one‑compartment model was used for the final model. While a two‑compartment model produced a 30.9‑point lower OFV (2266.85 vs 2297.73, prior to inclusion of any body‑size scalar), two‑compartment parameter estimates were unstable and imprecise, and visual inspection of concentration-time profiles on a log‑linear scale showed a predominantly linear decline consistent with one‑compartment disposition. In addition, previously published cephalexin PK analyses have uniformly used one‑compartment models, further supporting this choice.

*Absorption structure*: The transit model did not minimize successfully when the absorption rate constant (Ka), the transit rate constant (Ktr), and mean transit time (Mtt) were estimated simultaneously. Fixing Ka to Ktr (estimating only 2 transit parameters) reduced the OFV by 54.7 points (1928.16 vs 1982.89) relative to the final Tlag/Ka model. A two-parameter Weibull function also produced a significant OFV reduction, although slightly less than the transit model. Given the limited sampling in the absorption phase, both the transit and Weibull models produced high shrinkage for absorption parameters. For this reason, the simpler first‑order absorption model with Tlag was selected. A simpler model was also preferred because additional factors such as formulation (e.g., capsule vs suspension) or fed/fasting state likely influence absorption, but sample size limitations, especially during the absorption phase, prevented optimal evaluation of these covariates.

*Residual error*: A combined error model provided the best fit.

*BLQ Handling*: Alternative strategies did not improve model performance beyond Monolix’s native M4 method.

*Shrinkage and inter‑individual variability (IIV)*: IIV was initially included on Tlag, Ka, Vd/F, and CL/F. Estimates for Vd/F exhibited high eta shrinkage (53.8%), so random effects on Vd/F were fixed to zero to avoid over‑parameterization. Shrinkage values in the final model were acceptable for retained parameters: 25.1% for Tlag, 29.3% for Ka, and 20.6% for CL/F.

*Body size effects*: Inclusion of TBW or FFM on Vd/F and CL/F improved the model substantially (253- and 262-point OFV reductions, respectively, vs no size metric). FFM scaling was preferred over TBW (4.7-point lower OFV) after adding PMA on CL/F; this is physiologically plausible given cephalexin’s hydrophilicity and distribution primarily to extracellular water, which scales more closely with FFM, and FFM’s good correlation with renal maturation. Of note, FFM outperformed TBW only when the adult dataset (Study #4) was included. When the pediatric datasets (Studies #1-3) were evaluated separately, FFM and TBW performed similarly.

*Allometry*: Because the classic fixed allometric exponents (0.75 for CL/F, 1.0 for Vd/F) were originally derived for TBW, we allowed these exponents to be estimated when using FFM. The model successfully estimated values close to typical allometric expectations (0.82 for CL/F, 0.92 for Vd/F), and these estimated values were retained in the final model.

*PMA/eGFR effects on CL/F*: PMA inclusion on CL/F—as a Hill function with estimated Hill coefficient and TM_50_—was the most significant covariate after FFM inclusion, producing a 57-point OFV reduction and improving IIV of clearance estimates from 31% to 17% (CV%). The effect of PMA was more significant than eGFR (34.5-point OFV reduction). Adding eGFR to CL/F in addition to PMA reduced OFV by only 1.2 points and was not retained.

*PNA effects on Ka*: PNA was a significant covariate on Ka in the neonatal subset but did not appear significant in older subjects based on stratified plots of individual parameter estimates versus covariates. However, limited absorption‑phase sampling prevented more complex absorption structures. A Tlag/Ka absorption model was used in the final model, with PNA incorporated as a categorical covariate (9.8-point OFV reduction) affecting only subjects with PNA ≤21 days, applied as a scalar on the population Ka.

*Other Covariates*: No additional covariates were significant after incorporating size-based (FFM on V/F and CL/F) and age-based (PMA on CL/F and PNA on Ka) effects.

*Sensitivity Analysis of Influential Data*: During model development, three subjects—14 (from Study 1), 115 (from Study 2), and 210 (from Study 3)—were flagged as potential outliers given observations with population-weighted residuals greater than or less than 3. Parameter estimation and the covariate search process were re-performed after sequentially removing these outlier patients one at a time and with removal of all three. Removal of these influential subjects, assessed individually or in combination, did not meaningfully alter the parameter estimates or the statistical significance of covariate-parameter relationships, so they were retained in the final analysis. In addition, because Study 3 included subjects sampled at steady state, we also assessed its influence by re-estimating the final model after excluding all Study 3 subjects. Compared with the final model, this produced similar parameter estimates, covariate effects, and random effects, suggesting that inclusion of the steady-state dataset did not materially influence the final parameter estimates.

*Final structural and covariate-adjusted equations*:

$$\boldsymbol{Tlag}_{\boldsymbol{indiv}}={Tlag}_{pop}\cdot e^{\eta_{Tlag}}$$

$$PNA \leq21 days: \boldsymbol{Ka}_{\boldsymbol{indiv, \leq21}\boldsymbol{d PNA}}={Ka}_{pop}\cdot e^{\beta_{PNA}}\cdot e^{\eta_{Ka}}$$

$$PNA>21 days: \boldsymbol{Ka}_{\boldsymbol{indiv, >21}\boldsymbol{d PNA}}={Ka}_{pop}\cdot e^{\eta_{Ka}}$$

$$\frac{\boldsymbol{Vd}_{\boldsymbol{indiv}}}{\boldsymbol{F}}=\frac{{Vd}_{pop}}{F}\cdot\left( \frac{FFM}{56 kg} \right)^{0.92}$$

$$\frac{\boldsymbol{CL}_{\boldsymbol{indiv}}}{\boldsymbol{F}}=\frac{{CL}_{pop}}{F}\cdot\left( \frac{FFM}{56 kg} \right)^{0.82}\cdot\frac{{PMA}^{5.06}}{{42.96}^{5.06}+{PMA}^{5.06}}\cdot e^{\eta_{CL}}$$

where *indiv* and *pop* represent the individual and typical population values, respectively, for a parameter, *e* is a constant (base of the natural logarithm), η is the deviation from the typical population PK parameter value, and β_PNA_ is the fixed scalar of postnatal age on Ka.

**Appendix D:** Excluded Data

Prior to data analysis, the following data were excluded:

- **Study 1**: The original dataset from Study 1 included 34 subjects who contributed 149 cephalexin plasma concentrations. In the original analysis, one subject was excluded due to emesis occurring within 30 minutes of dosing (4 concentrations). Additionally, one physiologically implausible concentration was excluded during data review, resulting in an analyzed dataset of 144 concentrations from 33 subjects. The same analyzed dataset was used in this analysis.
- **Study 2**: No subjects or individual observations were excluded from Study 2.
- **Study 3**: No subjects or individual observations were excluded from Study 3.
- **Study 4**: Only observations obtained after receipt of cephalexin were included in the analyzed dataset. In Study 4, pre-dose samples were collected from all subjects, all of which were BLQ, and these were excluded.

No subjects or individual observations were excluded during data analysis (see above).

**Appendix E:** Probability of Target Attainment and Cumulative Fractional Response

**Methicillin-susceptible *Staphylococcus aureus***

*MIC distribution*: For MSSA, an MIC distribution utilized by USCAST found a cephalexin MIC_90_ = 8 mg/L, with nearly all isolates having MICs ≤16 mg/L, though only 55% of isolates having MICs ≤4 mg/L.^8^ We previously conducted a pediatric-specific MIC study and found a more favorable MIC distribution, with an MIC_90_ = 4 mg/L.^9^ We measured cephalexin MICs, in duplicate, for 48 clinical isolates from children with invasive osteoarticular infections and/or bacteremia that were identified as MSSA by clinical testing. All isolates had oxacillin MICs ≤0.5 mg/L and corresponding cephalexin MICs within 1-4 mg/L (21% with MIC=1, 52% with MIC=2, and 27% with MIC=4 mg/L). There is likely some geographic variability, as a U.S. study including isolates mainly from Texas also found a higher proportion of isolates with MIC = 8 mg/L.^10^ Given this discrepancy, we report CFR calculations for both MIC distributions.

*Pharmacodynamic Target*: USCAST proposes a PD target for MSSA, citing murine thigh infection model targets of 26% *f*T>MIC for net bacterial stasis and 34% *f*T>MIC for 1‑log kill.^8^ Consistent with this 1-log kill target, we used a primary PD target of 35% *f*T>MIC (rounding to the nearest 5% threshold).

*Susceptibility Breakpoints*: EUCAST recommends that susceptibility to cephalexin can be inferred for *S. aureus* isolates that are phenotypically (cefoxitin susceptible) or genotypically negative for mecA/mecC (e.g., MSSA).^11^ However, this classification is limited to treatment of “less severe infections” or “as oral follow-on therapy”, and that, if reported, cephalexin susceptibility should be reported as “susceptible, increased exposure” (EUCAST’s “intermediate” category). USCAST does report a cephalexin breakpoint for *S. aureus* (≤8 mg/L susceptible), but notes that this breakpoint is based on a net bacterial stasis endpoint and is only applicable for “non-severe, uncomplicated infections”.^12^ CLSI does not have specific breakpoints for cephalexin, though defines MSSA similar to the other agencies.

**Enterobacterales**

*MIC distribution*: For Enterobacterales, we limited our analysis to MICs ≤16 mg/L, as being reflective of Enterobacterales isolates with a known cefazolin MIC ≤2 mg/L, based on a previously published CLSI dataset of 205 Enterobacterales isolates (including *E. coli, K. pneumoniae, P. mirabilis*) tested by broth microdilution.^13^ In these data, the subset of Enterobacterales isolates with a cefazolin MIC ≤2 mg/L had corresponding cephalexin MICs of 4 mg/L (7% of isolates), 8 mg/L (84%), and 16 mg/L (9%), with none of those isolates having cephalexin MICs >16 mg/L. A cefazolin MIC ≤16 mg/L corresponded to cephalexin MICs of 4 mg/L (4.8% of isolates), 8 mg/L (66.3%), 16 mg/L (25.9%), and >16 mg/L (3.0%). Because reliably achieving high *f*T>MIC goals for MICs >16 mg/L is not feasible in most age groups, we used a cephalexin MIC distribution that corresponds with the more favorable cefazolin MIC of ≤2 mg/L for our primary CFR outcome. Data from EUCAST and USCAST are largely similar. EUCAST reports an ECOFF of 32, with 92% of isolates having MICs ≤16 mg/L (as of March 2026). USCAST reports a more resistant wild-type MIC distribution, with an MIC_90_ >512 mg/L and only 81% of isolates having MICs ≤16 mg/L.^8^

*Pharmacodynamic Target*: The only cephalexin-specific PD data come from a neutropenic murine thigh PD model in which net bacterial stasis was achieved for only 3 of 5 *E. coli* isolates (at 34%, 43%, and 61% *f*T>MIC) and l-log kill was achieved for only 2 of the 5 isolates (at 48% and 53% *f*T>MIC).^8^ After imputing net bacterial stasis of at least >61% *f*T>MIC for the isolates that did not meet the endpoint, USCAST used 61% *f*T>MIC as the stasis target. They also concluded that 1‑log kill could not be reliably achieved, though ~50% *f*T>MIC produced 1‑log kill in the isolates that did achieve this endpoint.

*Susceptibility Breakpoints*: Per CLSI, cefazolin can be used as a surrogate for predicting the activity of oral cephalosporins (including cephalexin) for uncomplicated UTIs due to *E. coli*, *K. pneumoniae*, and *P. mirabilis* using a breakpoint of ≤16 mg/L when using typical adult cephalexin doses (250-1000 mg q6 hours).^14^ These data were based on ≥97.1% categorical agreement using a cefazolin MIC of ≤16 mg/L based on the MIC distribution cited above.^13^ While cefazolin breakpoints for systemic Enterobacterales infections (e.g., those other than uncomplicated UTI) are ≤2 mg/L (susceptible), 4 mg/L (intermediate), and ≥8 mg/L (resistant), CLSI does not recommend using cefazolin surrogacy to predict cephalexin susceptibility for systemic infections. Similarly, EUCAST reports a cephalexin susceptibility breakpoint of ≤16 mg/L for uncomplicated UTI only.^15^ USCAST recommends against a cephalexin breakpoint for Enterobacterales, saying that the available evidence does not support its use for any systemic Enterobacterales infection, as their highest evaluated dose (1000 mg q6 hrs) did not achieve their net bacterial stasis target.^12^ No agency (i.e., CLSI, EUCAST, USCAST) considers cephalexin dosing above the FDA-labeled doses.

**Supplementary Tables and Figures**

**Table S1.** Reported adult cephalexin exposure following a single 1,000 mg oral dose across published studies. Adult cephalexin area under the plasma concentration-time curve from time zero extrapolated to infinity (AUC_0-inf_) values were compiled from published studies and used to characterize reference adult drug exposure. Reported values are shown with the corresponding standard deviation (SD), cephalexin dose and formulation, and feeding status from each study. When a study evaluated multiple formulations or feeding conditions, each reported condition is presented as a separate row. The final row reports the unweighted mean AUC_0-inf_ and SD across all included observations; each row contributed equally to the summary with adjustment for study sample size. These published adult exposure data were used to benchmark the reference adult cephalexin exposure for comparison with model-based pediatric simulations. NA, not applicable.

**Figure S1.** Individual observed concentration-time profiles (blue points) with corresponding model‑predicted individual fits (purple lines) for all 71 subjects. The vertical axis represents cephalexin concentration (mg/L), and the horizontal axis represents time after the first recorded dose (h). Subject IDs above each panel indicate study origin: 1-59 (Study 1), 101-117 (Study 2), 203-215 (Study 3), and 301-311 (Study 4). Dosing times are not displayed, but subjects with multiple concentration peaks were sampled following more than one cephalexin dose.

**Table S2.** Individual empirical Bayesian estimates for pharmacokinetic parameters by age group. Individual post hoc empirical Bayesian estimates of pharmacokinetic parameters are summarized for the overall population and by FDA-defined age group: 7-28 days, 29 days to <2 years, 2 to <12 years, 12 to <18 years, and 18 to 30 years. Parameters include absorption lag time (Tlag), first-order absorption rate constant (Ka), clearance (CL), volume of distribution (V), and elimination half-life. CL is presented as absolute clearance (L/h), normalized to fat-free mass (CL/FFM; L/h/kg FFM), normalized to total body weight (CL/kg; L/h/kg TBW), and standardized to a reference FFM of 56 kg using an allometric exponent of 0.82. Vd is presented as absolute volume (L), normalized to total body weight (V/kg; L/kg TBW), and standardized to a reference FFM of 56 kg using an allometric exponent of 0.92. Half-life was calculated as 0.693 × V/CL. Values are presented as median (range).

**Figure S2.** Relationship between absorption rate constant (Ka, 1/hr) and postnatal age (PNA, either days or years) from the base model. The base model includes allometric scaling of FFM on Vd/F and CL/F without additional covariate effects added. A linear trendline illustrates the increase in Ka with increasing PNA in neonates, but not in older children/adults.

**Figure S3.** Sigmoidal relationship between individual clearance (displayed as the percentage of adult CL value) and post-menstrual age (PMA, weeks). Solid line depicts the population-predicted maturation curve. Individual estimates are in blue.

**Figure S4.** Relationship between individual weight-normalized apparent clearance (CL/F) in L/hr/kg and postnatal age (in years, plotted on log scale). Individual estimates in blue; LOESS-smoothed trend across subjects in orange.

25 mg/kg (max 1,000 mg) q8 hrs

q8 hrs

1,000 mg q6 hrs

q8 hrs

25 mg/kg q12 hrs

q8 hrs

**Figure S5.** Simulated steady‑state AUC_0-24,SS_ for each pediatric age group, with 7-60‑day infants and 2-12‑month children pooled. Each virtual subject group received the AUC‑matched regimen corresponding to the adult 1,000 mg q6 hr dose: 1,000 mg q6 hr for adolescents (12-18 yr), 25 mg/kg (max 1,000 mg) q8 hr for children and infants >2 months, and 25 mg/kg q12 hrs for young infants. The red dashed line indicates the adult reference AUC_0-24,SS_ of 269.84 µg·h/mL. The dark gray shaded band represents ±20% of the adult reference AUC, and the light gray shaded band represents ±30% of the adult reference AUC.

**Table S3**. Probability of target attainment (PTA) results for three dosing regimens: 25 mg/kg (max 1,000 mg) q8 hr, 25 mg/kg (max 1,000 mg) q6 hrs, and 37 mg/kg (max 1,500 mg) q6 hrs. Each table block, stacked vertically, summarizes PTA by MIC for various *f*T>MIC targets, including 25% and 35% for 25 mg/kg (max 1,000 mg) q8 hr and 35%, 40%, 50%, and 60% *f*T>MIC for the higher dosing regimens. Age groups are pooled to simplify neonatal and infant strata. Values represent simulated PTAs at steady state for each regimen and age group.

**Figure S6.** CFR estimates for each cephalexin dose regimen and age group evaluated across pharmacodynamic (PD) targets ranging from 20% to 100% *f*T>MIC against methicillin-susceptible *Staphylococcus aureus* (using the USCAST MIC distribution, MIC_90_ = 8 mg/L). The horizontal red dotted line indicates the 90% CFR threshold. Vertical dashed lines indicate PD targets of 25% *f*T>MIC (gray, reflecting bacterial stasis) and 35% *f*T>MIC (black, reflecting 1-log kill). CFR, cumulative fraction of response; MIC, minimum inhibitory concentration.

**Figure S7**. CFR estimates for each cephalexin dose regimen and age group evaluated across pharmacodynamic (PD) targets ranging from 20% to 100% *f*T>MIC against Enterobacterales (for cefazolin MICs ≤2 mg/L). The horizontal red dotted line indicates the 90% CFR threshold. Vertical dashed lines indicate PD targets of 40%, 50%, and 60% *f*T>MIC. CFR, cumulative fraction of response; MIC, minimum inhibitory concentration.

**Table S4.** Highest *f*T>MIC target associated with an Enterobacterales CFR ≥90% (for the subset of isolates with a cefazolin MIC ≤2 mg/L) across dosing regimens and age groups. Values shown as <20% indicate that CFR was <90% even at the lowest target evaluated (20% *f*T>MIC). Cells with ‘n/a’ indicate that the regimen was not evaluated for that age group.

**References**

1.	Peters AM, Snelling HL, Glass DM, Bird NJ. Estimation of lean body mass in children. Br J Anaesth. 2011;106(5):719–23. doi: 10.1093/bja/aer057. PubMed PMID: 21498495.

2.	Al-Sallami HS, Goulding A, Grant A, Taylor R, Holford N, Duffull SB. Prediction of Fat-Free Mass in Children. Clin Pharmacokinet. 2015;54(11):1169–78. doi: 10.1007/s40262-015-0277-z. PubMed PMID: 25940825.

3.	Janmahasatian S, Duffull SB, Ash S, Ward LC, Byrne NM, Green B. Quantification of lean bodyweight. Clin Pharmacokinet. 2005;44(10):1051–65. doi: 10.2165/00003088-200544100-00004. PubMed PMID: 16176118.

4.	O'Hanlon CJ, Holford N, Sumpter A, Al-Sallami HS. Consistent methods for fat-free mass, creatinine clearance, and glomerular filtration rate to describe renal function from neonates to adults. CPT Pharmacometrics Syst Pharmacol. 2023;12(3):401–12. Epub 20230207. doi: 10.1002/psp4.12924. PubMed PMID: 36691877; PMCID: PMC10014044.

5.	Pierce CB, Munoz A, Ng DK, Warady BA, Furth SL, Schwartz GJ. Age- and sex-dependent clinical equations to estimate glomerular filtration rates in children and young adults with chronic kidney disease. Kidney Int. 2021;99(4):948–56. Epub 20201208. doi: 10.1016/j.kint.2020.10.047. PubMed PMID: 33301749; PMCID: PMC9083470.

6.	Smeets NJL, IntHout J, van der Burgh MJP, Schwartz GJ, Schreuder MF, de Wildt SN. Maturation of GFR in Term-Born Neonates: An Individual Participant Data Meta-Analysis. J Am Soc Nephrol. 2022;33(7):1277–92. Epub 20220426. doi: 10.1681/ASN.2021101326. PubMed PMID: 35474022; PMCID: PMC9257816.

7.	Smeets NJL, Teunissen EMM, van der Velden K, van der Burgh MJP, Linders DE, Teesselink E, Moes DAR, Tondel C, Ter Heine R, van Heijst A, Schreuder MF, de Wildt SN. Glomerular filtration rate in critically ill neonates and children: creatinine-based estimations versus iohexol-based measurements. Pediatr Nephrol. 2023;38(4):1087–97. Epub 20220802. doi: 10.1007/s00467-022-05651-w. PubMed PMID: 35916956; PMCID: PMC9925555.

8.	Andes D, Lepak A, Ambrose P, Bhavnani S, Rubino C, Sader H. Development of Modernized Susceptibility Guidance For Oral Cephalosporin Antimicrobial Agents Using Pharmacometric Approaches: United States Committee on Antimicrobial Susceptibility Testing (USCAST); 2024 [updated Dec 2024]. Available from: <https://drive.google.com/file/d/1P9Qp8ocSoPEy4mqdQkOp6MaJ7rhuCv_I/view>.

9.	Haynes AS, Prinzi A, Silveira LJ, Parker SK, Lampe JN, Kavanaugh JS, Horswill AR, Fish D. Cefadroxil Comparable to Cephalexin: Minimum Inhibitory Concentrations among Methicillin-Susceptible Staphylococcus aureus Isolates from Pediatric Musculoskeletal Infections. Microbiol Spectr. 2022;10(4):e0103922. Epub 2022/06/23. doi: 10.1128/spectrum.01039-22. PubMed PMID: 35730963; PMCID: PMC9431593.

10.	McNeil JC, Sommer LM, Boyle M, Hogan P, Vallejo JG, Hulten KG, Flores AR, Kaplan SL, Fritz S. Cefazolin Inoculum Effect and Methicillin-Susceptible Staphylococcus aureus Osteoarticular Infections in Children. Antimicrob Agents Chemother. 2020;64(9). Epub 20200820. doi: 10.1128/AAC.00703-20. PubMed PMID: 32660989; PMCID: PMC7449214.

11.	European Committee on Antimicrobial Susceptibility Testing. EUCAST Guidance Document on Cephalosporins for S. aureus Infection 2025 [updated 24 February 2025]. Available from: <https://www.eucast.org/fileadmin/eucast/pdf/guidance_documents/Cephalosporins_for_Staphylococcus_aureus_Infections_20250224.pdf>.

12.	United States Committee on Antimicrobial Susceptibility Testing. 2025 USCAST Interpretive Tables 2025. Available from: <https://www.uscast.org/breakpoints>.

13.	Schuetz AN, Brasso WB, Crandon JL, Hardy DJ, Jenkins SG, Jones RN, Knapp CC, Reller LB, Ad Hoc Oral Cephalosporin Working Group of the Subcommittee on Antimicrobial Susceptibility Testing of the C. Cefazolin as a class representative for oral cephalosporins and uncomplicated urinary tract infections caused by indicated Enterobacteriaceae. Diagn Microbiol Infect Dis. 2013;77(4):381–2. Epub 2013/10/05. doi: 10.1016/j.diagmicrobio.2013.08.024. PubMed PMID: 24091106.

14.	Lewis II JS. CLSI M100-ED35:2025 Performance Standards for Antimicrobial Susceptibility Testing, 32nd Edition. 35 ed: CLSI (Clinical and Laboratory Standards Institute); 2025.

15.	The European Committee on Antimicrobial Susceptibility Testing. Breakpoint tables for interpretation of MICs and zone diameters. Version 16.0, 2026. Available from: <https://www.eucast.org>.

**Supplementary Alt Text**

**Figure S1. Individual model fits**

Alt text:

Individual cephalexin concentration-time profiles for all subjects with observed data points and corresponding model‑predicted individual fits, separated by subject, to illustrate goodness of individual model fit.

**Figure S2. Absorption versus post‑natal age**

Alt text:

Scatter plot showing absorption rate constant versus post‑natal age, split between neonates ≤60 days and all older ages. A rising trend is evident in early infancy, with minimal age‑related change in absorption in older children and young adults.

**Figure S3. Clearance maturation based on post-menstrual age**

Alt text:

Plot illustrating the relationship between apparent clearance and post‑menstrual age on a logarithmic scale. Individual estimates are shown with a sigmoidal population maturation curve, demonstrating increasing clearance approaching adult values.

**Figure S4. Weight‑normalized clearance**

Alt text:

Scatter plot of weight‑normalized apparent clearance versus post‑natal age on a logarithmic scale, showing low weight-normalized clearance in early infancy, higher values in early childhood, and decreasing values in adolescents and young adults.

**Figure S5. AUC matching across age groups**

Alt text:

Boxplots of simulated steady‑state 24‑hour area under the curve (AUC) for pediatric age groups receiving exposure‑matched dosing regimens, compared with the adult reference exposure from 1,000‑mg dosing every six hours, overlayed with AUC values for the enrolled subjects receiving the same exposure-matched doses.

**Figure S6. CFR across pharmacodynamic targets for MSSA**

Alt text:

Line plots showing cumulative fractional response across pharmacodynamic targets from 20% to 100% free time above MIC, stratified by age group and cephalexin dosing regimen, for the treatment of methicillin-susceptible *Staphylococcus aureus*.

**Figure S7. CFR across pharmacodynamic targets for Enterobacterales**

Alt text:

Line plots showing cumulative fractional response across pharmacodynamic targets from 20% to 100% free time above MIC, stratified by age group and cephalexin dosing regimen, for the treatment of Enterobacterales.
